# Trajectories of cognitive and perceived functional decline in people with dementia: Findings from the IDEAL programme

**DOI:** 10.1002/alz.13448

**Published:** 2023-09-02

**Authors:** Anthony Martyr, Madhumathi Ravi, Laura D. Gamble, Robin G Morris, Jennifer M. Rusted, Claire Pentecost, Fiona E. Matthews, Linda Clare, IDEAL study team

**Affiliations:** ^1^ Centre for Research in Ageing and Cognitive Health University of Exeter Medical School St Luke's Campus Exeter UK; ^2^ Population Health Sciences Institute Newcastle University Newcastle upon Tyne Exeter UK; ^3^ Department of Psychology King's College London Institute of Psychiatry Psychology and Neuroscience London UK; ^4^ School of Psychology University of Sussex Falmer UK; ^5^ NIHR Applied Research Collaboration South‐West Peninsula Exeter UK

**Keywords:** Alzheimer's disease, instrumental activities of daily living, longitudinal, memory, rating accuracy

## Abstract

**INTRODUCTION:**

Impaired cognition and instrumental activities of daily living (iADL) are key diagnostic features of dementia; however, few studies have compared trajectories of cognition and iADL.

**METHODS:**

Participants from the IDEAL study comprised 1537, 1183, and 851 people with dementia, and 1277, 977, and 749 caregivers at baseline, 12 and 24 months, respectively. Addenbrooke's Cognitive Examination‐III and Functional Activities Questionnaire were used to measure cognition and iADL, respectively. Scores were converted to deciles.

**RESULTS:**

Self‐rated iADL declined on average by ‐0.08 (‐0.25, 0.08) decile points per timepoint more than cognition. Informant‐rated iADL declined on average by ‐0.31 (‐0.43, ‐0.18) decile points per timepoint more than cognition.

**DISCUSSION:**

Cognition and self‐rated iADL declined at a similar rate. Informant‐rated iADL declined at a significantly greater rate than cognition. Therefore, either cognition and perceived iADL decline at different rates or informants overestimate increasing iADL difficulties compared to both cognition and self‐ratings.

**Highlights:**

- Self‐ratings of the degree of functional difficulties were consistent with cognition
- Decline in self‐rated everyday activities was consistent with cognitive decline
- Informant‐ratings of everyday activities declined more than cognition

## 1 BACKGROUND

Impairment in both cognition and instrumental activities of daily living (iADL) is a key diagnostic feature of dementia, also termed major neurocognitive disorder.^1^, ^2^ Cognition is an umbrella term that encompasses different mental abilities such as memory, executive function, and attention, with iADL referring to the more complex everyday activities that require multiple cognitive processes.^3^ Research has reported a consistent association between these two aspects in people with dementia,^4^, ^5^, ^6^ both declining as the condition progresses.^7^, ^8^, ^9^, ^10^

Whilst cognition in people with dementia is measured using neuropsychological tests, there are different ways of measuring iADL in people with mild‐to‐moderate dementia.^4^, ^11^ The most frequent method is to ask informants, typically informal caregivers including family or friends, to rate perceived ability using a structured questionnaire.^4^, ^11^ Another method, is for people with dementia to rate their own iADL ability, although this is less widely used clinically because of corresponding difficulties in awareness of disability; ratings of iADL by people with dementia tend to indicate fewer difficulties than ratings by their caregivers.^12^, ^13^, ^14^, ^15^ This discrepancy is also associated with mood, caregiver stress, and the age of the person with dementia.^3^, ^10^, ^16^, ^17^, ^18^, ^19^

Few studies have compared trajectories of cognition and iADL in people with dementia to determine whether cognition and iADL decline at the same rate. The individual cognitive domains most frequently associated with iADL decline are executive function^7^, ^8^ and memory.^7^ However, global cognition, as measured by screening tests like the Addenbrooke's Cognitive Examination‐III (ACE‐III)^20^ has a stronger association with iADL than either memory or executive function.^4^, ^5^, ^10^ Prior to a dementia diagnosis, subtle cognitive and iADL difficulties develop at approximately the same time with estimated trajectories showing a roughly equivalent decrease in ability for cognition and iADL.^21^, ^22^ Indeed, studies that have separately investigated trajectories of cognition and iADL have shown that trajectories are similar over approximately 2 years.^12^, ^13^, ^23^ No study to our knowledge has directly compared rate of decline or similarity of trajectories in cognition and iADL in people with dementia.

The present study uses data from the Improving the experience of Dementia and Enhancing Active Life (IDEAL) study.^24^ The aim is to compare trajectories of cognition with self‐rated and informant‐rated iADL ability in a large sample of people with dementia. The study will investigate whether declines in perceived iADL are concordant with declines in cognition, or whether cognition declines at a faster or slower rate compared to self‐rated and/or informant‐rated iADL ability.

## 2 METHODS

### 2.1 Design

The IDEAL longitudinal cohort study of people with dementia and their caregivers in Britain^24^, ^25^ ran between 2014 and 2021. This paper presents longitudinal data using version 7 of the datasets from Time 1 (T1), Time 2 (T2), and Time 3 (T3). Initial assessments were conducted between July 2014 and August 2016; T2 assessments were conducted after 1 year (2015‐2017), and T3 after a further year (2016‐2018). Time 4 to Time 6 assessments were conducted between August 2018 and December 2021 but are not reported here as the relevant measures of cognition or iADL (see below) were not administered at these timepoints. The cohort at T1 comprised 1537 people with dementia together with 1277 caregivers, mostly spouses/partners. Caregivers provided informant ratings. People with dementia were recruited through UK National Health Service research networks across England, Scotland, and Wales. To meet inclusion criteria at entry to the study, participants had to have a clinical diagnosis of dementia as judged by clinicians at recruitment sites, a score of 15 or above on the Mini‐Mental State Examination (MMSE)^26^ indicating mild‐to‐moderate dementia, and the ability to communicate verbally in English. Exclusion criteria at T1 were co‐morbid terminal illness and inability to provide informed consent, and at any timepoint any known potential for home visits to pose a significant risk to researchers. Caregivers were recruited into the study if they were willing to take part and were providing regular care to the person with dementia.^27^ Full criteria for exclusion and consent are provided in the protocol.^24^ The IDEAL study was approved by the Wales Research Ethics Committee 5 (reference 13/WA/0405) and the Ethics Committee of the School of Psychology, Bangor University (reference 2014‐11684), and is registered with UKCRN, registration number 16593.

**RESEARCH IN CONTEXT**

1. **Systematic review**: PubMed was used to identify studies that investigated longitudinal changes in cognition and instrumental activities of daily living. Several publications were identified, and these studies were appropriately cited. No study was found that had compared trajectories in the same sample.
2. **Interpretation**: The results show that as difficulties with cognition increased, difficulties with both self‐rated and informant‐rated instrumental activities of daily living also increased. The trajectory over the two years suggests that informant‐rated instrumental activities of daily living declined at a significantly greater rate than cognition. Self‐rated instrumental activities of daily living declined at the same rate as cognition.
3. **Future directions**: These findings provide justification to further investigate the accuracy of self‐rated and informant‐rated instrumental activities of daily living over time, especially in relation to changes in cognition. More research is needed to compare perceived instrumental activities of daily living with objective performance.

### 2.2 Measures

Cognition of people with dementia was assessed with the ACE‐III; this has a score range of 0–100 and higher scores indicate better cognitive functioning. At T2 and T3 the ACE‐III was not administered if the person with dementia scored nine or lower on the MMSE; people with dementia scoring below 10 on the MMSE were administered the Test For Severe Impairment instead.^28^ Given that both ACE‐III and MMSE measure cognitive function and are strongly correlated (*r* = 0.72 at T1, *r* = 0.83 at T2, and *r* = 0.85 at T3) the MMSE score was used to predict and impute the ACE‐III score for these people. In the overall dataset, 25 ACE‐III scores were imputed at T2 and 55 were imputed at T3.

iADL ability was assessed with an 11‐item Functional Activities Questionnaire (FAQ);^29^ this was modified from the original FAQ by including a question concerning telephone use as described previously.^15^, ^17^, ^18^, ^19^ Each item was rated on a 0 to 3 scale leading to a score range of 0–33; a higher score indicated greater perceived difficulty with iADL abilities. Both self‐rated (FAQ‐S) and informant‐rated (FAQ‐I) versions were used in the study. People with dementia without a participating caregiver were included in the analyses using FAQ‐S ratings but not in the analyses using FAQ‐I ratings.

As mood of the person with dementia, caregiver stress, and background variables such as age of the person with dementia have previously been associated with iADL ratings^3^, ^10^, ^16^, ^17^, ^18^, ^19^ these were included as covariates. The Geriatric Depression Scale‐10 (GDS‐10)^30^ was used to measure depression in people with dementia, with higher scores indicating more self‐rated depressive symptoms. For the purposes of the analysis the sample was split into two groups: not depressed (GDS‐10 = 0‐3) and depressed (GDS‐10 = 4‐10). The Relatives’ Stress Scale^31^ was used to measure the level of self‐reported caregiver stress; possible scores range from 0 to 60 with higher scores indicating greater caregiver stress. Sociodemographic and diagnostic variables for the FAQ‐S analysis were person with dementia age, sex, level of education (no qualifications, school leaving certificate at age 16, school leaving certificate at age 18, university), and dementia diagnosis. For the FAQ‐I analysis informant age, sex, and caregiver status (spouse/partner, other) were also included. Dementia diagnosis was taken from medical records.

### 2.3 Procedure

The people with dementia and caregivers were visited at home by a researcher. At T1 participants were visited on three occasions spread over a few weeks, whereas at T2 and T3 participants were visited twice. Researchers administered the measures to people with dementia whereas caregivers completed the measures by themselves. Informed consent was obtained from both the person with dementia and from the caregiver (where available).

### 2.4 Planned analyses

Analysis was conducted using Stata v17. Regression was used to investigate change in ACE‐III and FAQ scores per timepoint using mixed effects models. After checking residuals for normality, a linear model was used for the ACE‐III and a generalized linear mixed model was used for the FAQ. To investigate associations between the ACE‐III and the FAQ at T1 the ACE‐III was regressed on the FAQ. To enable comparison of trajectories of the two measures, because of the differences in the scale and distribution of the ACE‐III and FAQ, scores were converted to deciles at T1 and these were imposed on scores at T2 and T3, resulting in a scale of 1 to 10 for both measures. Neither the ACE‐III nor the self‐rated or informant‐rated FAQ had floor or ceiling effects that would affect how the deciles were computed. For converted scores, a lower decile point indicated poorer performance. Mixed effects models with an interaction term were used to investigate whether there were differences in the rate of decline of the ACE‐III and the FAQ‐S or the FAQ‐I. First, an unadjusted model was conducted. The first adjusted model for the FAQ‐S included mean centered age, sex, level of education, and dementia diagnosis as covariates; for the FAQ‐I caregiver mean centered age, sex, and caregiver status were also included. In the final adjusted models for the FAQ‐S self‐rated GDS‐10 scores were added to the model, whereas for the FAQ‐I Relatives’ Stress Scale scores were added to the model. For analyses using educational level and dementia diagnosis scores on ACE‐III and FAQ‐S were converted to quintiles rather than deciles due to the smaller numbers in the subgroups. To account for missing data on outcomes (ACE‐III and FAQ), full information maximum likelihood estimation was used.

## 3 RESULTS

The background characteristics of the sample are described in Table 1. Briefly, just over half of people with dementia were diagnosed with Alzheimer's disease. People with dementia were on average 7 years older than their caregivers and were primarily related to their caregivers by marriage; 17% had no caregiver taking part in the study. A third of people with dementia met the cutoff for being depressed.

**TABLE 1:** Characteristics of the people with dementia and caregivers at T1, T2, and T3.

	People with dementia				Caregivers		
Characteristics	**T1 (*n* = 1537)**	**T2 (*n* = 1183)**	**T3 (*n* = 851)**		**T1 (*n* = 1266)**	**T2 (*n* = 977)**	**T3 (*n* = 749)**
Mean age (mean, SD)	76.40 (8.56)	77.18 (8.40)	77.52 (8.44)	Mean age (mean, SD)	69.13 (11.00)	70.19 (10.61)	70.82 (10.47)
Age groups n (%)				Age groups n (%)			
<65	134 (8.7)	89 (7.5)	66 (7.8)	<65	362 (28.6)	256 (26.2)	170 (22.7)
65‐69	177 (11.5)	129 (10.9)	72 (8.5)	65‐69	206 (16.3)	145 (14.8)	121 (16.2)
70‐74	258 (16.8)	193 (16.3)	159 (18.7)	70‐74	264 (20.9)	211 (21.6)	170 (22.7)
75‐79	366 (23.8)	268 (22.7)	172 (20.2)	75‐79	223 (17.6)	180 (18.4)	132 (17.6)
80+	602 (39.2)	504 (42.6)	382 (44.9)	80+	211 (16.7)	182 (18.6)	156 (20.8)
Missing	–	–	–	Missing	–	3 (0.3)	–
Sex n (%)				Sex n (%)			
Female	672 (43.7)	514 (43.4)	375 (44.1)	Female	878 (69.4)	675 (69.1)	512 (68.4)
Male	865 (56.3)	669 (56.6)	476 (55.9)	Male	388 (30.6)	302 (30.9)	237 (31.6)
Educational level n (%)				Educational level n (%)			
No qualifications	429 (27.9)	318 (26.9)	232 (27.3)	No qualifications	271 (21.4)	199 (20.4)	150 (20.0)
School leaving certificate at age 16	272 (17.7)	198 (16.7)	136 (16.0)	School leaving certificate at age 16	281 (22.2)	221 (22.6)	165 (22.0)
School leaving certificate at age 18	519 (33.8)	411 (34.7)	296 (34.8)	School leaving certificate at age 18	378 (29.9)	278 (28.5)	215 (28.7)
University	311 (20.2)	248 (21.0)	182 (21.4)	University	329 (26.0)	266 (27.2)	209 (27.9)
Missing	6 (0.4)	8 (0.7)	5 (0.6)	Missing	7 (0.6)	13 (1.3)	10 (1.3)
Dementia diagnosis n (%)				Diagnosis of the person with dementia n (%)^a^			
Alzheimer's disease	851 (55.4)	661 (55.9)	488 (57.3)	Alzheimer's disease	705 (55.7)	543 (55.6)	429 (57.3)
Vascular dementia	170 (11.1)	116 (9.8)	82 (9.6)	Vascular dementia	141 (11.1)	93 (9.5)	73 (9.7)
Mixed (Alzheimer's disease and vascular dementia)	324 (21.1)	264 (22.3)	185 (21.7)	Mixed (Alzheimer's disease and vascular dementia)	260 (20.5)	218 (22.3)	158 (21.1)
Frontotemporal dementia	54 (3.5)	40 (3.4)	32 (3.8)	Frontotemporal dementia	45 (3.6)	37 (3.8)	30 (4.0)
Parkinson's disease dementia	44 (2.9)	34 (2.9)	17 (2.0)	Parkinson's disease dementia	42 (3.3)	32 (3.3)	20 (2.7)
Dementia with Lewy bodies	53 (3.4)	39 (3.3)	27 (3.2)	Dementia with Lewy bodies	42 (3.3)	33 (3.4)	22 (2.9)
Unspecified/Other	41 (2.7)	29 (2.5)	20 (2.4)	Unspecified/Other	31 (2.4)	21 (2.1)	17 (2.3)
Caregiver status n (%)				Caregiver status n (%)			
Spouse/partner	1042 (67.8)	799 (67.5)	584 (68.6)	Spouse/partner	1035 (81.8)	817 (83.6)	628 (83.8)
Family/friend	235 (15.3)	165 (13.9)	111 (13.0)	Family/friend	231 (18.2)	160 (16.4)	121 (16.2)
No caregiver involved	260 (16.9)	219 (18.5)	156 (18.3)	No caregiver involved	–	–	–
GDS‐10 binary score n (%)				Mean stress total score (mean, SD)	19.15 (9.82)	21.70 (10.09)	22.97 (10.15)
Not depressed	1044 (67.9)	845 (71.4)	609 (71.6)	Missing n (%)	84 (6.6)	71 (7.3)	50 (6.7)
Depressed	447 (29.1)	300 (25.4)	202 (23.7)				
Missing n (%)	46 (3.0)	38 (3.2)	40 (4.7)				

Abbreviations: GDS‐10, Geriatric Depression Scale‐10; Stress, Relatives' Stress Scale.

^a^ Where the person with dementia has withdrawn from the study the last known diagnosis (e.g., the diagnosis recorded at the last timepoint where they took part) was used for 24 caregivers at T2, and 64 caregivers at T3.

The mean scores for each timepoint for the ACE‐III and the FAQ are described in Table 2. Mean scores at T1 are provided for those that dropped out of the study at T2, and mean scores at T2 are provided for those that dropped out of the study at T3. For both the ACE‐III and the FAQ, scores showed fewer cognitive and iADL difficulties for people that remained in the study compared to those that dropped out. The results in Table 3a indicate over the 2 years of the study that ACE‐III scores declined by approximately five points per year. For iADL, FAQ‐S scores were consistently lower than FAQ‐I scores, with both sets of scores increasing over time; see Table 3a. This indicates that people with dementia and their caregivers rated iADL ability as decreasing over the 2 years, reflected in increases in measurement scores; this increase was greater for the FAQ‐I with the FAQ‐S and FAQ‐I difference growing from 8.29 points at T1 to 10.68 points at T3; see Table 2. Trajectories of FAQ decile converted scores were compared for people with dementia with participating caregivers only and FAQ‐I declined by a quarter of a decile point more per year than FAQ‐S (FAQ‐S*FAQ‐I interaction estimate: −0.24, 95% CI −0.37, −0.11).

**TABLE 2:** Mean instrumental activities of daily living and cognition data at T1, T2, and T3.

	Time 1	Time 2	Time 3	Time 1 scores		Time 2 scores	
People with dementia	*n* = 1537	*n* = 1183	*n* = 851	Did not take part at Time 2	Took part at Time 2	Did not take part at Time 3	Took part at Time 3
Functional ability							
Mean FAQ‐S total score (mean, SD)	9.61 (7.69)	11.13 (8.39)	12.26 (9.01)	11.14 (8.17)	9.14 (7.48)	13.33 (8.98)	10.32 (8.01)
Missing n (%)	54 (3.5)	181 (15.3)	113 (13.3)				
Cognition							
Mean ACE‐III total score (mean, SD)^a^	68.55 (13.52)	64.42 (16.88)	61.70 (20.40)	63.65 (13.40)	69.97 (13.23)	58.07 (17.83)	68.06 (15.73)
Missing n (%)	38 (2.5)	77 (6.5)	59 (6.9)				

	Time 1	Time 2	Time 3				
Caregivers	*n* = 1266	*n* = 977	*n* = 749				
Functional ability of the person with dementia							
Mean FAQ‐I total score (mean, SD)	17.90 (8.61)	20.91 (8.58)	22.94 (8.70)	19.63 (8.69)	17.38 (8.52)	23.12 (7.96)	20.17 (8.67)
Missing n (%)	94 (7.4)	43 (4.4)	25 (3.3)				
Cognition of the person with dementia							
Mean ACE‐III total score (mean, SD)^a^	68.23 (13.82)	64.61 (17.16)	60.41 (20.90)	64.94 (14.07)	69.27 (13.58)	59.05 (17.69)	66.36 (16.61)
Missing n (%)	27 (2.1)	75 (7.7)	107 (14.3)				

Abbreviations: ACE‐III, Addenbrooke's Cognitive Examination‐III; FAQ‐I, informant‐rated Functional Activities Questionnaire; FAQ‐S, self‐rated Functional Activities Questionnaire.

^a^ ACE‐III Time 2 and Time 3 data includes imputed scores for people whose Mini‐Mental State Examination score was below 10.

**TABLE 3:** Modeling the relationship between the ACE‐III and the FAQ at Time 1 and longitudinally.

a) Change in ACE‐III and FAQ per timepoint			
	Unadjusted estimate (95% CI)	Adjusted^a^ estimate (95% CI)	
ACE‐III (linear mixed model)	−5.45 (−5.91, −5.00)	−6.41 (−7.47, −5.35)	

	Unadjusted rate ratio (95% CI)	Adjusted^c^ rate ratio (95% CI)	
FAQ‐S (generalized linear mixed model)	1.18 (1.15, 1.22)	1.26 (1.18, 1.34)	
FAQ‐I (generalized linear mixed model)	1.18 (1.16, 1.20)	1.22 (1.16, 1.08)	

b) Association of FAQ and ACE‐III scores at Time 1			
People with dementia	Unadjusted Estimate (95% CI)	Model 1^a^ Estimate (95% CI)	Model 2^b^ Estimate (95% CI)
FAQ‐S on ACE‐III	−0.75 (−0.83, −0.67)	−0.76 (−0.84, −0.68)	−0.83 (−0.92, −0.74)

	Unadjusted Estimate (95% CI)	Model 1^c^ Estimate (95% CI)	Model 2^d^ Estimate (95% CI)
FAQ‐I on ACE‐III	−0.79 (−0.85, −0.69)	−0.79 (−0.87, −0.71)	−0.81 (−0.90, −0.72)

c) Difference in the trajectories of ACE‐III and FAQ decile points			
People with dementia	Unadjusted Estimate (95% CI)	Model 1^a^ Estimate (95% CI)	Model 2^b^ Estimate (95% CI)
ACE‐III*FAQ‐S interaction	−0.08 (−0.25, 0.08)	−0.07 (−0.24, 0.09)	−0.07 (−0.23, 0.10)

Caregivers			
	Unadjusted Estimate (95% CI)	Model 1^c^ Estimate (95% CI)	Model 2^d^ Estimate (95% CI)
ACE‐III*FFAQ‐I interaction	−0.31 (−0.43, −0.18)	−0.31 (−0.43, −0.18)	−0.30 (−0.42, −0.17)

Abbreviations: ACE‐III, Addenbrooke's Cognitive Examination‐III; FAQ‐I, informant‐rated Functional Activities Questionnaire; FAQ‐S, self‐rated Functional Activities Questionnaire.

^a^ Adjusted for person with dementia sex, age, level of education, and diagnosis type.

^b^ Adjusted for person with dementia sex, age, level of education, diagnosis, and depression.

^c^ Adjusted for person with dementia and caregiver sex, person with dementia and caregiver age, caregiver status, person with dementia diagnosis, and person with dementia level of education.

^d^ Adjusted for person with dementia and caregiver sex, person with dementia and caregiver age, caregiver status, person with dementia diagnosis, person with dementia level of education, and caregiver stress.

### 3.1 Self‐rated iADL ability and cognition

There was a negative relationship between ACE‐III and FAQ‐S; see Table 3b. This suggests that people with dementia with more cognitive difficulties rated themselves as having greater iADL difficulties. This relationship was strengthened after adjusting for background characteristics of the person with dementia, level of education, dementia diagnosis, and depression.

**FIGURE 1:**
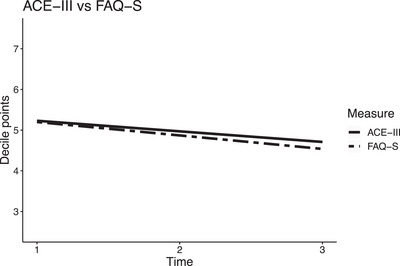
Person with dementia ACE‐III and FAQ‐S over time, Adjusted model 2. ACE‐III, Addenbrooke's Cognitive Examination‐III; FAQ‐S, self‐rated Functional Activities Questionnaire.

When comparing the trajectories of decile rank converted FAQ‐S and ACE‐III scores, in the unadjusted model, FAQ‐S declined on average by an additional 0.08 decile points per timepoint compared to ACE‐III; see Table 3c. This minimal difference remained similar after adjusting for person with dementia sociodemographic and diagnostic variables and when further adjusting for depression. The trajectory over the 2 years suggests that FAQ‐S and ACE‐III ratings declined at a similar rate; see Figure 1.

Trajectories were compared for different dementia diagnoses; see Table 4. As with the overall sample, FAQ‐S and ACE‐III declined at a similar rate for all dementia subtypes except for frontotemporal dementia, where FAQ‐S declined at a greater rate than ACE‐III. Trajectories were also compared according to educational level; see Table 5. FAQ‐S and ACE‐III declined at a similar rate for all groups except those who left school at 18 years where FAQ‐S declined at a slightly greater rate than ACE‐III; however, confidence intervals overlapped between the groups suggesting no difference.

**TABLE 4:** Difference in trajectories of ACE‐III and FAQ‐S quintile points for different dementia subtypes.

	Unadjusted	Model 1^a^	Model 2^b^
ACE‐III*FAQ‐S interaction	Estimate (95% CI)	Estimate (95% CI)	Estimate (95% CI)
Alzheimer's disease	−0.10 (−0.21, 0.00)	−0.10 (−0.21, 0.01)	−0.10 (−0.20, 0.01)
Vascular dementia	−0.05 (−0.31, 0.23)	−0.05 (−0.30, 0.21)	−0.03 (−0.29, 0.22)
Mixed (Alzheimer's disease and vascular dementia)	−0.08 (−0.25, 0.09)	−0.07 (−0.24, 0.10)	−0.07 (−0.24, 0.10)
Frontotemporal dementia	−0.49 (−0.89, −0.09)	−0.50 (−0.90, −0.09)	−0.40 (−0.81, 0.00)
Parkinson's disease dementia	−0.14 (−0.66, 0.37)	−0.10 (−0.59, 0.40)	−0.19 (−0.69, 0.31)
Dementia with Lewy bodies	−0.11 (−0.57, 0.36)	−0.04 (−0.51, 0.42)	−0.05 (−0.53, 0.41)
Unspecified/Other	−0.09 (−0.58, 0.41)	−0.09 (−0.58, 0.41)	−0.08 (−0.58, 0.42)

Abbreviations: ACE‐III, Addenbrooke's Cognitive Examination‐III; FAQ‐S, self‐rated Functional Activities Questionnaire.

^a^ Adjusted for person with dementia sex, age, and level of education.

^b^ Adjusted for person with dementia sex, age, level of education, and depression.

**TABLE 5:** Difference in trajectories of ACE‐III and FAQ‐S quintile points for subgroups with different levels of education.

	Unadjusted	Model 1^a^	Model 2^b^
ACE‐III*FAQ‐S interaction	Estimate (95% CI)	Estimate (95% CI)	Estimate (95% CI)
No qualifications	−0.06 (−0.21, 0.09)	−0.06 (−0.21, 0.10)	−0.06 (−0.21, 0.10)
School leaving certificate at age 16	−0.05 (−0.24, 0.15)	−0.05 (−0.24, 0.14)	−0.05 (−0.24, 0.15)
School leaving certificate at age 18	−0.13 (−0.26, 0.00)	−0.13 (−0.26, 0.00)	−0.11 (−0.24, 0.02)
University	0.03 (−0.15, 0.21)	0.03 (−0.15, 0.20)	0.02 (−0.16, 0.20)

Abbreviations: ACE‐III, Addenbrooke's Cognitive Examination‐III; FAQ‐S, self‐rated Functional Activities Questionnaire.

^a^ Adjusted for person with dementia sex, age, and dementia diagnosis.

^b^ Adjusted for person with dementia sex, age, dementia diagnosis, and depression.

### 3.2 Informant‐rated iADL ability and cognition

As shown in Table 3b, at T1 there was a negative relationship between ACE‐III and FAQ‐I suggesting that people with dementia with more cognitive difficulties were rated by their caregivers as having greater iADL difficulties. The relationship remained similar following adjustment for person with dementia and caregiver sociodemographic and diagnostic variables and further adjustment for caregiver stress.

**FIGURE 2 alz13448-fig-0002:**
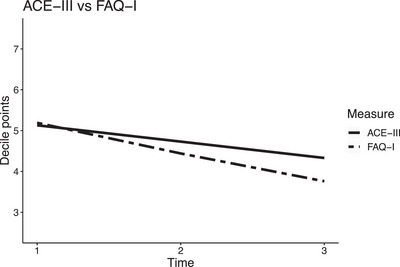
Person with dementia ACE‐III and caregiver FAQ‐I over time, Adjusted model 2. ACE‐III, Addenbrooke's Cognitive Examination‐III; FAQ‐I, informant‐rated Functional Activities Questionnaire.

When comparing the trajectories of decile rank converted FAQ‐I and ACE‐III scores, the slope of FAQ‐I declined on average by a further 0.31 decile points per timepoint compared to ACE‐III; see Table 3c. When adjusting for person with dementia and caregiver sociodemographic and diagnostic variables, the slope of FAQ‐I was still steeper than ACE‐III and remained similar with further adjustment for caregiver stress. The trajectory over the 2 years suggests that FAQ‐I ratings declined at a greater rate than ACE‐III scores; see Figure 2.

## 4 DISCUSSION

This is the first study, to our knowledge, to directly compare trajectories of cognition and iADL in a large sample of people with mild‐to‐moderate dementia. Cognition and self‐rated and informant‐rated iADL declined over the 2 years of the study; and this decline was unrelated to dementia diagnosis and level of education. Ratings indicated that perceived decline was greater in informant‐rated than self‐rated iADL. Trajectory analysis suggested that changes in informant‐rated iADL were greater than concomitant changes in cognition, whereas self‐rated iADL declined at a similar rate to changes in cognition; this was despite both self‐rated and informant‐rated iADL having a similar relationship with cognition at T1. This suggests that over the 2 years of the study informant‐rated iADL changes seem to diverge from the trajectory of how cognition changes and indicates that caregivers perceive more iADL difficulties as time progresses; therefore, either cognition and iADL decline at different rates or caregivers overestimate increases in iADL difficulties compared to both cognition and self‐ratings.

The findings are consistent with a growing body of research which suggests that over time people with dementia acknowledge their declining ability to perform everyday tasks.^12^, ^13^ This calls into question the widespread belief that people with mild‐to‐moderate dementia are not able to provide reliable iADL ratings due to their cognitive difficulties.^32^, ^33^, ^34^, ^35^ Indeed, in the present study, people with dementia with more cognitive difficulties rated themselves as having more iADL difficulties than people with fewer cognitive difficulties, and the vast majority rated their iADL ability as impaired, that is, their scores exceeded the FAQ cutoff that indicates impairment.^29^ In addition, a study that used self‐rated iADL scores reported that while self‐ratings were significantly lower than corresponding informant ratings, nearly two‐thirds exceeded the cutoff for impairment.^15^ This suggests that a majority of people with mild‐to‐moderate dementia perceive iADL difficulties as impaired.[Fig alz13448-fig-0002]

Findings are also consistent with earlier studies where generally people with dementia recognize that they have difficulties with iADL.^12^, ^14^, ^15^, ^17^, ^18^, ^36^, ^37^ Findings, however, are also consistent with self‐rated iADL reflecting fewer difficulties than informant‐rated iADL.^12^, ^13^, ^14^, ^15^, ^18^, ^37^ One possible explanation for this discrepancy could be that people with dementia may be unable to update their mental store or ‘personal database’ of information about the self and hence adhere to an outdated estimation of their performance.^38^ There is convincing evidence for this impaired updating in the memory domain;^39^ however, iADL ratings by people with dementia generally indicate increasing difficulties over time,^12^, ^13^ suggesting that they are able to update their personal database for iADL tasks at least to some extent. When self‐rated iADL are compared with objective performance, people with dementia generally appraise their own functioning more accurately than ratings made by their caregivers.^36^ Indeed, caregivers typically overestimate difficulties compared with objective performance.^36^, ^40^ Thus people with mild‐to‐moderate dementia may be able to monitor performance of iADL tasks and update their personal database to acknowledge iADL difficulties.

That informant‐rated iADL showed greater decline over the 2 years than self‐rated iADL and cognition is broadly consistent with earlier studies.^12^, ^13^ This is important for interpreting clinical and research data as it suggests that factors other than cognition might be influencing how caregivers rate iADL. The factors influencing informant‐rated iADL should be further investigated as these factors could be affecting the reliability of informant‐rated iADL ratings over time. This is especially important considering that the difference between self‐rated and informant‐rated iADL in the present study grew wider when compared to cognition over the duration of the study. When compared with objective measures of iADL, previous studies have found that caregivers often overestimate difficulties.^36^, ^40^

A possible reason why caregivers rate iADL difficulties over time as increasing more markedly relative to cognitive decline and self‐rated iADL may relate to the effects of increasing adjustment to their caregiver role,^41^ that is, evolution of coping style and coping strategies, increasing caregiving experience, and changes in the types of care provided.^40^, ^42^, ^43^ As caregivers become accustomed to the demands of caregiving they might be more able to recognize or more willing to acknowledge difficulties. This might make it more likely for caregivers to rate iADL as more impaired than self‐ratings made by people with dementia. In mild dementia, many caregivers do not necessarily see themselves as providing care^44^ and they may be unaware of the amount of support they provide. As they begin to acknowledge their caregiving role, and comprehend the level of their care provision, over time they may take responsibility for doing tasks that the person may still be capable of doing in order to keep them safe.^44^ Therefore, future research studies could investigate with mixed methods how caregivers with varying levels of caregiving experience adjust to their caregiving role and how their evolving understanding of iADL difficulties in the person with dementia changes over time. This could comprise qualitative interviews at regular intervals and a brief objective measure of iADL with corresponding appraisal ratings; this would allow for a deeper understanding of how caregivers and people with dementia perceive iADL difficulties as well as help understand changes in caregiver adjustment, coping strategies, and stress/burden. This is especially salient as coping strategies, adjustment, and burden influence how informants rate iADL difficulties,^10^, ^40^, ^45^, ^46^, ^47^ and higher perceived stress is often associated with more perceived iADL difficulties.^3^, ^10^, ^15^, ^17^, ^19^

How ratings accord with actual abilities is unknown, though a previous study found that people with mild‐to‐moderate dementia were able to rate performance on iADL tasks more accurately than corresponding ratings by their caregivers, particularly with tasks that do not largely rely on intact memory ability; for example, telling the time, or identifying medication.^36^ Further research is needed to validate how ratings provided by both people with dementia and their caregivers relate to actual iADL difficulties. It would be important to investigate how self‐ratings and informant ratings compare with objectively assessed iADL abilities over time. This would elucidate whether iADL abilities remain consistently associated with cognition over time, as suggested in the present study by self‐rated iADL ability, or whether iADL abilities decline more than cognition, as suggested in the present study by informant‐rated iADL ability. This would also further inform estimated trajectories that propose a roughly equivalent decrease in iADL ability and cognition throughout the course of dementia.^21^, ^22^

This study has some limitations that need to be acknowledged. Employing questionnaires to assess iADL rather than objective assessments was a limitation since questionnaires only offer a perceived assessment of iADL abilities which can be prone to various biases including depression in people with dementia, caregiver stress, and the age of the person with dementia in informant ratings.^3^, ^10^, ^15^, ^16^, ^18^, ^19^ Objective assessments have their own limitations^48^ but investigating how changes in objectively‐assessed iADL compare to changes in scores on objective tests of cognition could allow for a more direct comparison between iADL and cognition and could make comparing trajectories more reliable. The proportion of spousal caregivers was higher compared with existing data.^49^ This limits the generalizability of the results to non‐spousal caregivers; although the analysis controlled for caregiver status and previously no difference in cross‐sectional FAQ score has been reported between spousal and other caregiver types.^15^, ^17^, ^18^ Another limitation was in handling the different scales and distributions of the ACE‐III and the FAQ measures. Participants were ranked and deciles created for both ACE‐III and FAQ based on T1 data. Score ranges were extracted for each decile, but there is some inaccuracy in fractionating the scores into 10 equal deciles for both measures, which is larger for FAQ given the smaller score range. Results were checked for consistency where a score straddled two deciles; allocation into either the higher or lower decile did not affect the findings. Another limitation is that people with more cognitive and/or iADL difficulties may have been more likely to have withdrawn from the study, or not been included in the analysis at later timepoints as they were unable to respond using the FAQ responses. Using mixed effects models with full information maximum likelihood estimation helps to mitigate this issue to some extent as people with either ACE‐III or FAQ scores at any timepoint were included in the study and calculation of the slope for each individual is based on all timepoints that are available.

This is the first study to our knowledge to compare trajectories of iADL and cognition in people with mild‐to‐moderate dementia over time. The findings suggest that self‐rated iADL abilities change over time in line with cognition whereas informant‐rated iADL diverges and indicates a perceived increase in difficulties compared to cognition. Therefore, the findings suggest that either cognition and perceived iADL decline at different rates, or caregivers overestimate increasing iADL difficulties compared to both cognition and self‐ratings. This is important for clinical assessments and research as it suggests that people with mild‐to‐moderate dementia are able to recognize and acknowledge difficulties in their ability to undertake everyday activities. Conversely, the findings also suggest that, when compared with cognition, caregivers may overestimate difficulties in iADL. Further research is needed to confirm trajectories using objective assessments of iADL as this will remove potential biases concerning the perceived nature of iADL ratings.

## AUTHOR CONTRIBUTIONS

Anthony Martyr ideated the research questions. Anthony Martyr, Madhumathi Ravi, and Laura D. Gamble drafted the article and interpreted the analysis. Laura D. Gamble is responsible for the data analysis under the supervision of Fiona E. Matthews. Anthony Martyr, Madhumathi Ravi, and Laura D. Gamble curated the IDEAL datasets. Anthony Martyr, Robin G. Morris, Jennifer M. Rusted, Fiona E. Matthews, and Linda Clare were involved in the original conception, design, and funding acquisition of the IDEAL programme. All authors contributed to the critical revision of the article and approved the version to be published.

## CONFLICTS OF INTEREST STATEMENT

The authors have no conflicts of interest to declare supporting information

## CONSENT STATEMENT

This study was conducted in accordance with the Declaration of Helsinki and the guidelines on good clinical practice. All eligible participants who had signed the consent form were included in the study. This study protocol was reviewed and approved by the Wales Research Ethics Committee 5 (reference 13/WA/0405) and the Ethics Committee of the School of Psychology, Bangor University (reference 2014‐11684). All participants gave written informed consent.

## Supporting information

Supporting Information

## Data Availability

IDEAL data were deposited with the UK Data Archive in April 2020. Details of how the data can be accessed can be found here: https://reshare.ukdataservice.ac.uk/854293/.
